# Supplementary material for: Biocomposite Hydrogels for the Treatment of Bacterial Infections: Physicochemical Characterization and In Vitro Assessment
Source: Pharmaceutics. 2021 Dec 4;13(12):2079. doi: 10.3390/pharmaceutics13122079 (PMC8706181; doi:10.3390/pharmaceutics13122079)
Supplement: Supplementary file 1 [file pharmaceutics-13-02079-s001.zip › pharmaceutics-1443487-supplementary.pdf]

Supplementary Materials

Biocomposite Hydrogels for the Treatment of Bacterial Infections: Physicochemical Characterization and In Vitro Assessment

Delia Mihaela Rata, Anca Niculina Cadinoiu, Marcel Popa, Leonard Ionut Atanase, Oana Maria Daraba, Irina Popescu, Laura Ecaterina Romila and Daniela Luminita Ichim

Table S1. EDAX elemental analysis for biocomposite hydrogels.

Sample	Element	Weight %	Atomic %
C2P4.10.Z3	C K	54.93	63.68
	N K	1.94	1.93
	O K	38.88	33.83
	**ZnK**	**1.53**	**0.38**
	PtM	2.72	0.18
C2P4.10.Z5	C K	48.42	57.45
	N K	3.82	3.89
	O K	42.4	37.76
	**ZnK**	**3.4**	**0.77**
	PtM	1.96	0.13
C2P4.15.Z3	C K	53.6	62.04
	N K	1.59	1.58
	O K	41.33	35.91
	**ZnK**	**1.57**	**0.33**
	PtM	1.91	0.14
C2P4.15.Z3	C K	49.21	58.91
	N K	3.16	3.24
	O K	40.87	36.73
	**ZnK**	**3.48**	**0.93**
	PtM	3.28	0.19

Table S2. ANOVA statistical test for evaluation of antibacterial activity.

**Anova statistical test on *S. aureus***					
	**Sum of Squares**	**Df**	**Mean Square**	**F-ratio**	**Significance Level**
Between groups	82.6875	1	82.6875	9.9661	0.0342
Within groups	33.1875	4	8.2968		
Total	115.875	5			
**Anova statistical test on *E. coli***					
	**Sum of Squares**	**Df**	**Mean Square**	**F-ratio**	**Significance Level**
Between groups	63.0208	1	63.0208	79.0850	0.0008
Within groups	3.1875	4	0.7968		
Total	66.2083	5			
**Anova statistical test on *K. pneumonia***					
	**Sum of Squares**	**Df**	**Mean Square**	**F-ratio**	**Significance Level**
Between groups	29.0785	1	29.0785	22.997	0.0086
Within groups	5.0578	4	1.2644		
Total	34.1363	5			
